# Exercise for advanced prostate cancer: a multicomponent, feasibility, trial protocol for men with metastatic castrate-resistant prostate cancer (EXACT)

**DOI:** 10.1186/s40814-019-0486-6

**Published:** 2019-08-16

**Authors:** Malcolm Brown, Marie Murphy, Lauri McDermott, Helen McAneney, Joe M. O’Sullivan, Suneil Jain, Gillian Prue

**Affiliations:** 1 School of Nursing & Midwifery, Medical Biology Centre, Queen’s University Belfast, Lisburn Road, Belfast, Northern Ireland BT9 7BL; 2 Sport & Exercise Sciences Research Institute, Ulster University, Jordanstown, Northern Ireland BT37 0QB; 3 Insight Centre for Data Analytics, Dublin City University, Glasnevin, Dublin 9 Ireland; 4 Centre for Public Health, Queen’s University Belfast, Belfast, Northern Ireland BT12 6BA; 5 Centre for Cancer Research & Cell Biology, Queen’s University Belfast, Belfast, Northern Ireland BT9 7AE

**Keywords:** Home-based exercise, Aerobic, Strength, Advanced prostate cancer, Behavioural change

## Abstract

**Background:**

Men with metastatic castrate-resistant prostate cancer can experience an array of treatment-related side effects. Accumulating evidence suggests exercise may alleviate some of these adversities and assist in disease management. However, empirical evidence in advanced prostate cancer patients remains limited. The purpose of this study is to determine whether men with metastatic prostate cancer, who are ineligible for high-intensity exercise, can partake in a home-based, moderate-intensity exercise program and the impact of doing so on quality of life and physical fitness parameters.

**Methods:**

Thirty men with adenocarcinoma of the prostate and progressive systemic, metastatic disease will be recruited. Clinicians will screen patients against inclusion criteria to determine eligibility. All men enrolled will be prescribed a tailored, home-based, moderate-intensity exercise intervention consisting of aerobic and strengthening components for 12 weeks. Patients will receive supplementary education materials and weekly behavioural change consultations throughout the intervention. The primary outcome will be the feasibility of delivering such an intervention in men with metastatic disease. Secondary endpoints including skeletal events will be monitored for safety, as will the feasibility of patient-reported outcome measures and the sampling time points, generating data pertaining to completion rates and potential effect in future trials. General physical fitness will be assessed during these visits, using timed sit-to-stand testing and a 6-min walking test. Prior to each visit, objective physical activity levels will be captured for 7 days using an accelerometer, to determine the feasibility of this technology and the quality of data obtained. In parallel with the feasibility aspects of the trial, changes compared to baseline will be reported. Direct regular contact will also serve as a feedback loop, should any issues arise. This study has received ethical approval from the Office for Research Ethics Committees Northern Ireland.

**Conclusions:**

This study aims to determine the potential utility of a home-based exercise intervention in managing side effects associated with advanced prostate cancer and its treatment. This feasibility trial will inform the design and implementation of a larger randomised control trial to determine the efficacy of moderate aerobic and strengthening exercise as an adjuvant therapy in men with metastatic prostate cancer. Collecting such evidence provides further support for exercise in this paradigm and potential for its inclusion as a low-toxicity therapy in standard cancer care, in the longer term.

**Trial registration:**

ClinicalTrials.gov, NCT03658486

Trial sponsor: Queen’s University Belfast (Reference: B18/15). Contact: Dr. Paula Tighe, Research and Enterprise, Queen’s University Belfast. Telephone: 02890 973,296. Email: p.tighe@qub.ac.uk. The sponsor reviewed the protocol and ethical application prior to submission.

Protocol issue: Version 1 (18th May 2018). Authors: MB, MM, SJ and GP.

## Introduction

The annual incidence rate for prostate cancer (PCa) in Northern Ireland is approximately 1100, making it the most prevalent cancer in males [1]. Of those diagnosed, 20% are advanced cases, characterised by metastatic progression to secondary sites and the ultimate development of resistance to hormone therapy [2]. Some patients enter the advanced disease state having initially failed primary treatment with radiotherapy or surgery. Commonly, metastatic PCa patients receive castration therapy, usually in the form of luteinising hormone-releasing hormone agonist (LHRHa) therapy. Despite significant developments, such methods can cause a number of adverse effects [3]. Numerous physical problems are presented (e.g. sexual dysfunction, urinary incontinence, reduced bone mineral density, increased fat mass and reduced muscle mass) while psychological issues such as anxiety and depression also arise [4]. Metastatic spread to the bone is common, subsequently increasing bone pain and fracture risk and shortening survival [5, 6]. Men with castrate-resistant, bone metastatic PCa may also receive chemotherapy, which can also cause adverse effects including severe fatigue [4]. Thus, the adverse effects of PCa and its treatment present a considerable clinical issue.

Accumulating evidence suggests that regular exercise can induce a host of physiological and psychological benefits, which may alleviate certain treatment-related toxicities and improve disease outcomes [7, 8]. Previous exercise research in cancer populations adds further credibility, to the extent that regular aerobic exercise may confer a risk reduction of fatality [9]. An initial study, in men receiving androgen deprivation therapy (ADT) for localised PCa, reported progressive resistance training increased muscular strength, endurance and physical function [10]. A follow-up combined exercise program reported improved lean muscle mass, muscular strength, aerobic fitness and quality of life (QOL) while ameliorating fatigue and inflammation [11]. Other clinical exercise studies also report lower risks in progression, mortality, cancer-related fatigue and pain [9, 12]. Consequently, tailored exercise has developed into a promising and effective therapy particularly in localised PCa [13]. However, the potential mechanism(s) remains a topic of debate but may be founded in endocrine regulation (insulin-like growth factor (IGF)-1 or testosterone signalling), metabolism, reactive oxygen species and antioxidant signalling, epigenetics or cytokine signalling among potential others [14, 15].

Despite early findings, advanced prostate patients are normally omitted due to their increased risk of fracture and hence do not avail of such health benefits. Cormie et al. [16] conducted a preliminary supervised resistance exercise program in metastatic patients and reported high retention rates alongside improved physical function and lean muscle mass compared to the control. These beneficial changes persisted at 6-month follow-up along with improvements in QOL [17]. The exercise volume, including intensity, appears a key mediator in accruing such health benefits and enhancing survival [9, 18]. This recent progress and international recognition has resulted in several position stands and expert statements endorsing exercise in cancer care including advanced cancers [19–23].

Though awareness is increasing, clinical and empirical evidence in PCa patients with bone metastases remains limited [16]. Appropriately designed and supervised training programs have proved feasible and clinically meaningful, but the chronic effects are yet to be determined [16]. The Movember GAP4 INTERVAL trial is seeking to determine such effects through a collaborative, randomised control trial [8]. We anticipate that some men with advanced disease may be ineligible or unable to tolerate the exercise component (i.e. high-intensity training), so we aim to provide a parallel intervention to ensure these men have the opportunity to capitalise on the benefits of partaking in regular exercise, albeit at moderate intensity. Our proposed study is the first to examine the effects of a progressive, home-based, combined moderate program, with continual behavioural support in this population. Research to date tends to utilise supervised exercise with good reason and success, but at this time an intensive model of exercise is potentially unsustainable in the UK National Health Service (i.e. cost and resource). Home-based exercise is emerging as a feasible and effective strategy for post-therapy cancer survivors [24] and a key component in future cancer trials [25]. Further, a recent small study of cancer survivors (*n* = 20 Australian adults) expressed a preference for this exercise option [26]; thus, the need to evaluate a remotely supervised regime in this population is necessary. This study aims to provide meaningful clinical information with the hope of directly impacting future care.

Our primary objective is to establish the feasibility of delivering an individualised, home-based walking and strengthening exercise intervention to men with advanced PCa, who are unable or ineligible for high-intensity exercise. The feasibility evaluation will address the accessibility and acceptability of the intervention as well as recruitment, attrition and exercise adherence rates. The secondary objectives will (1) determine the feasibility on QOL, functional ability and patient-reported assessments and any changes from baseline and (2) collect preliminary data on health-related QOL and health resource usage to inform cost-effectiveness. All men recruited will receive the intervention to refine and optimise the methodology. Whilst a randomised control trial (RCT) is the ‘gold standard’ for garnering evidence, supporting the putative role of exercise in cancer care, given how resource-intensive and challenging exercise trials are, we first aim to determine the efficacy and acceptability in a smaller feasibility study, to determine if and how the full trial should be conducted [27, 28]. Feasibility trials also improve the chances of conducting a high-quality RCT and inform methodological design and resource requirements, to reduce the likelihood of research waste [28, 29]. We propose this exercise intervention will measurably impact QOL in men with advanced PCa.

## Methods

### Study design

**Fig. 1:**
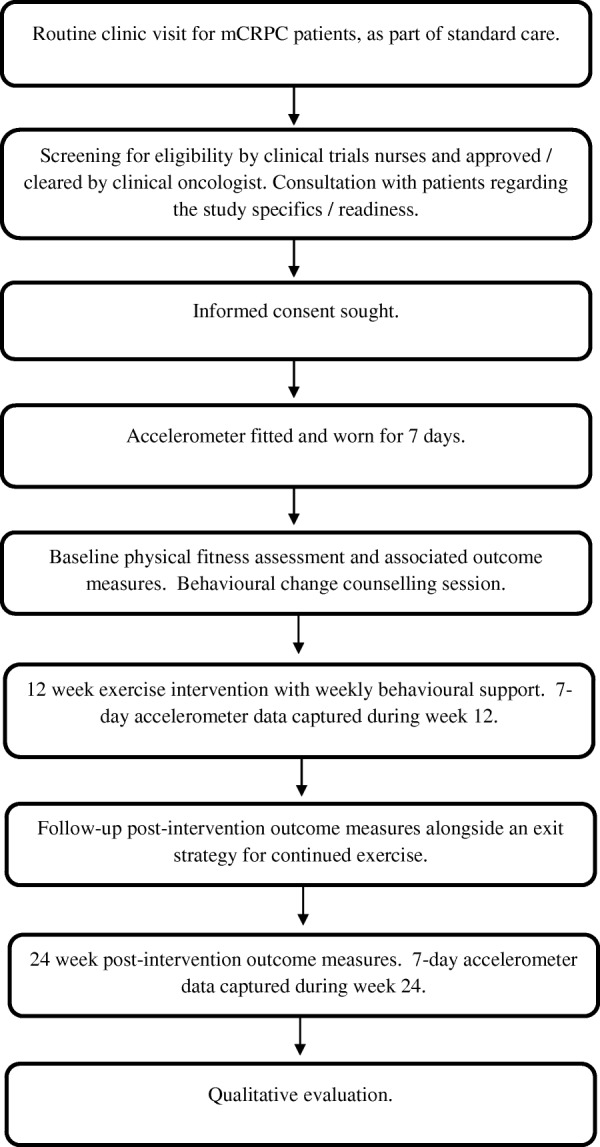
Study design and patient flow throughout the 24 weeks and follow-up

**Fig. 2:**
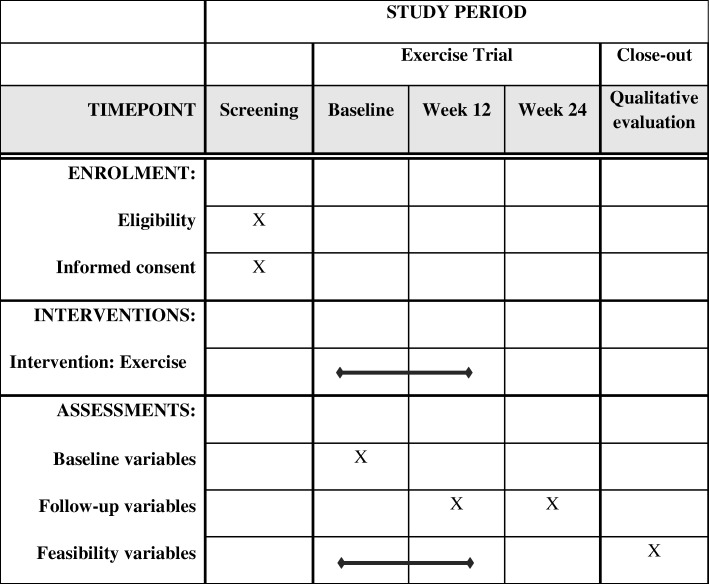
SPIRIT figure

This study is a single-arm, feasibility trial coordinated by Queen’s University Belfast in conjunction with the Northern Ireland Cancer Centre (Belfast Health and Social Care Trust). All patients will be assigned to the exercise intervention to establish feasibility and provide justification for a randomised control trial. This trial was registered on 5 September 2018 (https://clinicaltrials.gov/ct2/show/NCT03658486) and has since opened for recruitment. A chart detailing study procedures can be found in Fig. 1. This protocol has been developed using the Standard Protocol Items: Recommendations for Interventional Trials (SPIRIT) guidelines. Figure 2 shows the SPIRIT figure.

### Study objectives

The primary objective of this study is to establish the feasibility of delivering a prescribed exercise intervention to men with advanced prostate cancer, who are ineligible for high-intensity exercise (i.e. the Movember GAP4 INTERVAL study). As part of this primary objective, we will collect data:

- To determine patient eligibility and recruitment rates.
- To assess adherence to the programme and attrition rates.
- To determine the rate of exercise-induced adverse events (if any).
- To ascertain the extent to which the intervention might be integrated into clinical practice (and the cost implications of doing so).
- To refine methodological variables, optimising the study design and data collection processes.
- To explore the perceptions, acceptability and experiences of men with advanced prostate cancer undertaking the intervention and those men eligible that declined participation.
- To explore the perception and experience of those recruiting to and/or delivering the intervention (e.g. Exercise Physiologist/Clinical Oncologists/Research Nurses).

Secondary objectives will focus on the feasibility of data collection processes, mainly collecting changes in body composition, functional ability, physical fitness, physical activity levels and patient-reported outcome measures (cancer-related fatigue, pain and quality of life). Additionally, we will use this feasibility trial to collect preliminary data on health resource usage to inform cost-effectiveness of future trials and refine statistical considerations (e.g. ascertain sample size estimates).

### Progression criteria

Our methods for determining progression to a definitive RCT are based on recent recommendations [30]. Trial progression will primarily depend on the feasibility trial satisfying recruitment targets and secondarily protocol adherence targets, pre-determined by the trial steering committee. For example, 75–100% recruitment of the target sample size will enable progression to a main trial application, with little or no changes to relevant aspects of the protocol, while < 25% recruitment will prevent the trial from progressing. Recruitment of 50–74% will enable progression following a review of patients deemed ineligible or who declined the study invite, recruitment barriers and possible changes to relevant aspects of the protocol. Recruitment of 25–49% will only progress if a ‘rescue plan’ can be developed by the trial team and may involve identifying additional sites or changes to the protocol. Protocol adherence targets will be held to similar standards. Objectives relating to the feasibility of data collection will support the design of a definitive trial, but we have not assigned progression criteria.

### Participants

Men with adenocarcinoma of the prostate and progressive mCRPC will be recruited from the Northern Ireland Cancer Centre, Belfast. Patients must be on androgen deprivation therapy (ADT) with a gonadotropin-releasing hormone (GnRH) agonist/antagonist or have undergone bilateral orchiectomy. All patients must remain castrated throughout the study period. At enrolment, patients must be receiving abiraterone or enzalutamide, with no evidence of progression. Patients must also be ≥ 4 weeks since last surgery and fully recovered. At enrolment, patients must have no known contraindications to moderate-intensity exercise, including, but not limited to, acute congestive heart failure, unstable angina, recent myocardial infarction and peripheral neuropathy greater than or equal to grade 3. Men will be included if they have an Eastern Cooperative Oncology Group performance status of 0–2. Fluency and an understanding of English language is also a requirement due to the qualitative interviewing component. Lastly, men will be 18 years of age with no upper age limit for entry, and medical clearance to partake will be sought from their treating clinician. Exclusion criteria can be found in Table 1.

**Table 1:** Exclusion criteria

Men currently exceeding ACSM recommended exercise guidelines.	
Men with brain metastases.	
Men with a currently active second malignancy other than non-melanoma skin cancer.	
Congestive heart failure or recent serious cardiovascular event.	
Severe chest pain brought on by exercise.	

### Sample size calculation

As this is a feasibility study, a formal sample size calculation has not been performed. We aim to recruit 30 patients to the study which is standard practice for feasibility trials [31–33]. Assuming an 80% adherence rate, 25 patients should complete the intervention and subsequent evaluation.

### Screening

The clinical care team will screen clinic lists to determine eligibility and introduce the study researchers who can provide further information. While attending treatment clinics, clinical research nurses and members of the research team will screen each patient against the inclusion criteria. Following full disclosure of the study, informed consent will be obtained from each patient. Participants meeting current physical activity guidelines (≥ 150 min of moderate-intensity or ≥ 75 min of high-intensity exercise per week) at screening (determined via International Physical Activity Questionnaire and in consultation with an Exercise Physiologist) will be deemed suitably active and excluded. The remit of this study is to progressively accrue 150 min of moderate-intensity exercise weekly, so conceivably those already doing so have a higher exercise tolerance gained through a process of adaptation and are unlikely to gain the intended health benefits. Following medical clearance from a clinical oncologist, patients will be required to complete a series of baseline questionnaires and will undertake basic physical fitness and anthropometric assessments.

### Exercise prescription

This multicomponent, self-managed, home-based exercise strategy has been previously trialled in colorectal cancer patients (NCT02607787). The program will consist of 12 weeks of moderate-intensity walking (55–70% max HR; 12–14 Borg scale) and strengthening exercises (predominantly body weight; e.g. wall press, sit-to-stand; lateral raise and bicep curls, using household items for resistance). Brisk walking was selected as the mode of exercise for this feasibility trial as it poses a low risk of injury and due to its popularity, accessibility, cost-effectiveness and ease in adjusting exercise intensity/demand. Prior to each exercise session, participants will complete a warm up and at the end a cool down. Participants will receive a pedometer and an exercise booklet detailing the desired exercise prescription, allowing space to record daily exertion, particularly the mode, duration and intensity. Information to enable safe exercise, injury avoidance and overcoming barriers, as well as lifestyle strategies to incorporate exercise, will also be provided. The exercise program will be individually tailored and progressed over the 12-week duration, aiming to achieve recommended ACSM guidelines upon completion (Table 2). The individualised, self-managed nature of the program will allow for a level of autoregulation allowing modifications based on readiness and flexibility to complete certain exercises at a later date. A further advantage of autoregulation is that once patients become more active, they can work beyond their set program (e.g. increase walking distance or intensity). In the event that metastatic lesions pose a contraindication to certain exercises, the program will be modified and adapted. Participants will be required to attend the NICC to obtain outcome measures (QOL questionnaires, anthropometric and physical fitness testing) on three occasions (Table 3). The week prior to each of these visits, patients will wear an accelerometer to objectively record 7-day physical activity levels (at baseline, 12 and 24 weeks). Anthropometric measures will comprise height, weight, hip and waist circumferences while strength and cardiovascular endurance will be determined via a timed sit-to-stand test and 6-min walking test respectively. At 12 weeks, participants, in consultation with the exercise professional, will discuss a suitable exit strategy to enable exercise maintenance. All participants will be followed up at 24 weeks. Though the intervention is predominantly completed at home, weekly telephone contact permits a degree of remote supervision. An example of the exercise prescription, for an inactive patient at baseline, can be found in Table 4.

**Table 2:** Aerobic exercise goals at various time points during the intervention

Week	Aerobic exercise targets
3	Walk continuously for 10 min on at least 5 days of the week.
6	Walk continuously for 10 min, twice per day on at least 5 days of the week.
9	Walk continuously for 10 min, three times per day on at least 5 days of the week.
12	Progress to 30 min of brisk walking on at least 5 days of the week.

**Table 3:** Study assessment schedule

Outcome	Screening	Baseline	Weeks 1–12	Week 12	Week 24	Week 25
Eligibility						
Medical history	x	x	x	x	x	
Medication	x	x	x	x	x	
Medical clearance	x	x	x	x	x	
Anthropometric						
Height		x				
Weight		x		x	x	
Hip circumference		x		x	x	
Waist circumference		x		x	x	
Physical fitness						
Six-minute walking test		x		x	x	
Timed sit-to-stand test		x		x	x	
Patient-reported outcomes						
FACIT-fatigue		x		x	x	
FACT-P		x		x	x	
EQ5D		x		x	x	
BPI-SF		x		x	x	
Self-reported physical activity						
IPAQ-short form		x		x	x	
Objective physical activity						
Accelerometer assessment		x		x	x	
Behavioural support						
Weekly communication			x			
Feasibility						
Recruitment rates			x			x
Adherence			x			x
Attrition rates			x			x
Safety/adverse events			x			x
Patient experience			x			x

*Abbreviations*: *FACIT* Functional Assessment of Cancer Therapy, *FACT-P* Functional Assessment of Cancer Therapy—Prostate, *EQ5D* EuroQOL five-dimensional questionnaire, *BPI-SF* Brief Pain Inventory—Short Form, *IPAQ* International Physical Activity Questionnaire

**Table 4:** Weekly exercise prescription example

Week	Exercise prescription
1	AE: MIW for 10 min; once per day; at least 2 days of the week. STE: One set of 8–15 repetitions (2 exercises); on 2–3 days of the week.
2	AE: MIW for 10 min; once per day; at least 3 days of the week. STE: Two sets of 8–15 repetitions (2 exercises); on 2–3 days of the week.
3	AE: MIW for 10 min; once per day; at least 5 days of the week. STE: Three sets of 8–15 repetitions (2 exercises); on 2–3 days of the week.
4	AE: MIW for 10 min; once per day; at least 5 days per week (additional goal: on 2 or more days add another 1 × 10 min bout). STE: Same as week 3; add 1 set of 2 new exercises (4 total); 8–15 repetitions on 2–3 days.
5	AE: MIW for 10 min; once per day; at least 5 days of the week (additional goal: on 2 or more days add another 1 × 10 min bout). STE: Same as week 3; add 2 sets of 2 new exercises (4 total); 8–15 repetitions on 2–3 days.
6	AE: MIW for 10 min; twice per day; at least 5 days of the week (additional goal: on 2 or more days add another 1 × 10 min bout). STE: Same as week 3; add 3 sets of 2 new exercises (4 total); 8–15 repetitions on 2–3 days.
7	AE: MIW for 10 min; twice per day; at least 5 days of the week (additional goal: on 2 or more days add another 1 × 10 min bout). STE: Same as week 6; add 1 set of 2 new exercises (6 total); 8–15 repetitions on 2–3 days.
8	AE: MIW for 10 min; twice per day; at least 5 days of the week (additional goal: on 3 or more days add another 1 × 10 min bout). STE: Same as week 6; add 2 sets of 2 new exercises (6 total); 8–15 repetitions on 2–3 days.
9	AE: MIW for 10 min; twice per day; at least 5 days of the week (additional goal: on 3 or more days add another 1 × 10 min bout). STE: Same as week 6; add 3 sets of 2 new exercises (6 total); 8–15 repetitions on 2–3 days.
10–12 (maintenance)	AE: MIW for 30 min on at least 5 days of the week. STE: Same as week 9. For continued progression, add another set of each exercise.

*Abbreviations*: *AE* aerobic exercise, *MIW* moderate-intensity walking, *STE* strength training exercise

### Behavioural support

The behaviour change component of this particular programme is based on the COM-B method [34]. Behavioural support will comprise a behavioural change consultation at baseline and weekly, structured telephone contact. The behavioural support component will follow a scripted protocol of defined questioning, to maintain intervention fidelity. The same member of the research team will lead the behavioural consultations throughout, to maintain reliability. Information pertaining to weekly exercise adherence, perceived barriers and potential solutions, weekly goal setting and self-confidence will be recorded. Behavioural support sessions provide the opportunity to reinforce key aspects of the trial and a chance to provide positive feedback on progress. Records of call durations and patient availability will be stored. The behavioural support programme will ensure continuity in the exercise prescribed, so all patients receive equal support to meet the desired exercise volume by study cessation. The purpose of this communication is to maintain a close relationship with the patient and identify additional support required. Accurate recording of physical activity data is essential to enable associations with treatment-related adversities and the outcome markers. Participants will specify their preferred communication type (if not telephone), to ensure regular contact is maintained.

### Primary endpoint

The primary endpoint is to determine the feasibility of delivering a tailored, home-based, 12-week exercise intervention in men with metastatic castrate-resistant prostate cancer. Recruitment and attrition rates, exercise adherence (number of sessions completed), general safety/adverse events and the patient experience will be a focus of determining feasibility.

### Secondary endpoints

Secondary endpoints will be assessed at baseline, 12 and 24 weeks accounting for pain, cancer-related fatigue, skeletal-related events, physical fitness and QOL. Direct contact throughout the study will also serve as a feedback loop for certain measures should they occur outside each visit. Secondary outcome measures will be assessed mainly for completeness in accordance with the feasibility theme of the study. Although the sample size is small and perhaps underpowered to definitely report causation, changes from baseline will be analysed, to provide an indication of efficacy and detect any differences. It should be noted that all secondary outcome measures are obtained principally for testing the ability to undertake and complete measures and not to detect differences from baseline. Processes relating to data handling and analysis are described in greater detail in the “Data analysis” section.

#### Pain progression and opiate use

Pain and analgesic management using opiates will be assessed using the Brief Pain Inventory Short Form (BPI-SF) [35] and a review of medical records at baseline, 12 and 24 weeks. The BPI was originally developed to assess pain severity and impact in cancer patients, with reported good internal consistency and validity [36, 37]. The BPI-SF is now widely recommended as a core outcome measure in clinical trials.

#### Skeletal-related events

Symptomatic skeletal-related events (SSEs) will be determined at treatment clinics and reviewing patient medical records, continuously throughout the 24-week intervention.

#### Fatigue and QOL measures

Cancer-related fatigue will be determined using the FACIT fatigue scale at baseline, 12 and 24 weeks [38]. The Functional Assessment of Cancer Therapy—Prostate (FACT-P) and EuroQOL five-dimensional questionnaire (EQ-5D-5 L) will be measured at the same time points to assess QOL. Both questionnaires are internationally recognised validated measures for all stages of prostate cancer and are sensitive to changes in indicators of treatment efficacy [39–41]. The change in score for the FACT-P and FACIT fatigue questionnaires can be calculated for each participant and categorised according to pre-established minimally important differences (MIDs). The EQ-5D-5 L will be used as a general index of health status and to inform the cost-effectiveness of future trials across the five dimensions.

#### Physical activity levels

Each patient’s level of physical activity will be assessed subjectively at baseline, 12 and 24 weeks using the International Physical Activity Questionnaire Short Form (IPAQ-SF). Craig et al. [42] reported the IPAQ-SF produced repeatable data and has acceptable measurement properties for self-report physical activity. Lee and colleagues [43] have reported the IPAQ-SF typically overestimates physical activity and thus should be supplemented with objective measures. In order to prevent this potential overestimation, this questionnaire will be completed in consultation with an Exercise Physiologist. Further, to complement the subjective assessment, objective physical activity will be captured the week prior to each outcome visit, using an accelerometer (ActiGraph GT3X). The accelerometer will be worn for 7 days, capturing data at 10-s Epoch intervals. The analysis will be completed at 60-s Epoch intervals, in accordance with recommendations [44, 45]. Moderate to vigorous physical activity and step counts will be assessed for each patient.

#### Physical fitness

Physical fitness will be assessed at baseline, 12 and 24 weeks using a timed sit-to-stand test (30 s) and 6-min walking test. The number of repetitions will be recorded during the sit-to-stand test, while the distance covered will determine aerobic fitness.

### Qualitative evaluation

Upon study completion (week 24), the impact and experiences of key stakeholders including active participants, eligible patients that declined participation and the research staff delivering the intervention will be collected, via face-to-face semi-structured interview. Men who declined to participate will be given the opportunity to discuss their decision and gather information on suitable alternatives. Study staff interviews will be conducted by an impartial colleague to reduce bias.

The feasibility, acceptability, potential utility of the program, approaches to optimise and appropriateness of the timing and outcome measures will be a priority during interviewing. The semi-structured interviews will explore perceived facilitators and barriers to exercise and experiences of exercise in managing treatment-related symptoms, with common themes extracted during content analysis. Information relating to the number screened and eligible, retention and compliance, follow-up rates and satisfaction will be recorded on a discussed weekly but revisited during interviewing, followed by a descriptive analysis. Interviews will be tape-recorded and transcribed verbatim.

### Ethical approval

Ethical approval to conduct this study was obtained from the Office for Research Ethics Committees Northern Ireland (ORECNI) in 2018 (IRAS project ID: 248301). Any subsequent protocol modifications will be reviewed by ORECNI. Following approval, a memorandum, detailing the amendment, will be issued to all members of the trial steering committee.

### Data management and monitoring

All data collected will be stored securely in accordance with General Data Protection Regulations (within a key card accessed building, on a password-protected computer and locked filing cabinet). Only members of the study management team will have access to this data. A trial steering committee (comprising MB, MM, LM, HM, JMO, SJ, GP and NICRCF advisory group members) will oversee the trial and convene regularly to discuss pertinent aspects of the study. As clinical oncologists sit on this trial steering committee, we felt a trial data monitoring committee was not required. Spontaneous adverse events will be captured during regular, weekly contact with patients. Any adverse events will be expedited to the trial oncologists, upon report, for an immediate resolution and further reviewed at regular committee meetings. This trial will be subject to audit from the study sponsor, who has the power to terminate the trial if necessary. Trial results will be submitted for publication and communicated in a relevant medical or scientific journal. Anonymity will be maintained and unique identifiers will be removed in any subsequent outputs.

### Data analysis

From a feasibility perspective, data analysis will account for the number of patients screened, numbers participating in the intervention and the numbers unwilling to participate, after eligibility is confirmed, with reasons for non-participation. This data will be examined and scrutinised using descriptive analysis to identify differences between participants and non-participants. Patient compliance, utilisation and satisfaction with the intervention will be assessed, as will completion rates for the intervention and the outcome measures. The acceptability of the physical outcome measures and questionnaires used in determining health resource use will also be reported. Additionally, the feasibility of the accelerometer data (e.g. volume and quality) will be analysed. All measures will be scored according to standard practice and analysed for mean changes from baseline, using a one-way repeated measures ANOVA. For each outcome measure, change will be calculated as follow-up (at both 12 and 24 weeks) minus baseline and presented alongside 95% CIs. We will also calculate effect size (Cohen’s *d*) for each outcome measure, at 12 and 24 weeks, using the following formula: (mean post-test − mean baseline)/(baseline standard deviation). Conventions of small (*d* = 0.20), medium (*d* = 0.50) and large (*d* = 0.80) will be used. Preliminary feasibility results will inform the future RCT sample size calculation and the parameters for investigation to determine potential clinical meaningfulness.

Transcriptions of audio recorded interviews will be analysed using thematic analysis [46]. At each stage, findings will be verified and discussed in order to assess the accuracy of the interpretation, promote reliability and ensure rigour [47]. NVivo qualitative data analysis software will be used to aid data management. Data generated from interviewing will assist in determining the safety of the intervention as well as the health economics moving forward.

## Discussion

Accumulating evidence supports the putative synergistic role of exercise in managing the myriad of treatment-related adversities associated with prostate cancer [2]. Data from recent epidemiological studies provides further recognition of the association between exercise and a reduced risk of prostate cancer mortality [18, 48, 49]. Collectively, we now have level 1 evidence that exercise is effective in improving QOL, fatigue and exercise tolerance in men with prostate cancer, with higher quality results observed in advanced cases treated with ADT [7]. Commonly, due to the high-risk nature of advanced prostate cancer (i.e. heightened risk of fractures) as well as poor retention and compliance, supervised exercise is advocated and has proved effective. Yet, despite the reported benefits such a regime, at this time, is economically unsustainable in current NHS settings, due to the limited number of specialist exercise professionals, equipment and resources, highlighting a necessity for establishing an alternative. Culos-Reed and colleagues [50] successfully implemented a tailored, partly home-based intervention and reported an attenuation of the side effects associated with treatment. Recently, Hardcastle et al. [26] presented several key variables that influence exercise participation in cancer populations including availability, access, time, cost and confidence. A notable finding identified a keen interest in home-based, remotely supervised exercise alongside professional exercise counselling [26].

Implementing a remotely supervised walking and strengthening programme with sufficient, ongoing behavioural support is appealing and can overcome many perceived barriers, in terms of its practicality (incorporating into daily living) and cost-effectiveness (home-based with little investment in equipment or memberships). We hope this intervention caters for perceived patient preferences and will assist in uptake and adherence, but admittedly may prove resource intensive, with dedicated researcher time invested in weekly contact. Perhaps exercise provision for mCRPC patients could eventually be embedded in the standard of care follow-up consultations between patients and trained, specialist nurses/physiotherapists, if feasible, to alleviate some of this time burden, while resulting in a coordinated effort to facilitate behavioural change. This study will investigate whether men with advanced prostate cancer can take part in a home-based, progressive, moderate-intensity exercise program and its effect on treatment-related side effects. We propose that providing the necessary education and continual behavioural support will empower patients and enhance their self-efficacy, a key variable in maintenance. The potential utility of home-based exercise in assisting disease management is of clinical interest and warrants further scientific investigation, highlighting a rationale for the current study.

## Trial status

This feasibility trial commenced recruitment in December 2018 and is currently ongoing. At the time of submission of this protocol, seven patients have consented and entered the trial.

## Data Availability

Not applicable.
